# A new and effective two-step clustering approach for single cell RNA sequencing data

**DOI:** 10.1186/s12864-023-09577-x

**Published:** 2023-11-09

**Authors:** Ruiyi Li, Jihong Guan, Zhiye Wang, Shuigeng Zhou

**Affiliations:** 1 Translational Medical Center for Stem Cell Therapy, Shanghai East Hospital, and School of Medicine, Tongji University, 1239 Siping Road, 200092 Shanghai, China; 2 Department of Computer Science and Technology, Tongji University, 4800 Caoan Road, 201804 Shanghai, China; 3 Shanghai Key Lab of Intelligent Information Processing, and School of Computer Science, Fudan University, 2005 Songhu Road, 200438 Shanghai, China

**Keywords:** Single cell RNA sequencing, Random walk, Hierarchical clustering

## Abstract

**Background:**

The rapid devolvement of single cell RNA sequencing (scRNA-seq) technology leads to huge amounts of scRNA-seq data, which greatly advance the research of many biomedical fields involving tissue heterogeneity, pathogenesis of disease and drug resistance etc. One major task in scRNA-seq data analysis is to cluster cells in terms of their expression characteristics. Up to now, a number of methods have been proposed to infer cell clusters, yet there is still much space to improve their performance.

**Results:**

In this paper, we develop a new two-step clustering approach to effectively cluster scRNA-seq data, which is called *TSC* — the abbreviation of *T*wo-*S*tep *C*lustering. Particularly, by dividing all cells into two types: core cells (those possibly lying around the centers of clusters) and non-core cells (those locating in the boundary areas of clusters), we first clusters the core cells by hierarchical clustering (*the first step*) and then assigns the non-core cells to the corresponding nearest clusters (*the second step*). Extensive experiments on 12 real scRNA-seq datasets show that *TSC* outperforms the state of the art methods.

**Conclusion:**

TSC is an effective clustering method due to its two-steps clustering strategy, and it is a useful tool for scRNA-seq data analysis.

**Supplementary Information:**

The online version contains supplementary material available at 10.1186/s12864-023-09577-x.

## Background

As the basic structural and functional units of all known organisms, cells vary broadly in types and states [1]. Assessing cell-to-cell variability in expression is crucial for disentangling heterogeneous tissues and understanding dynamic biological processes [2]. In traditional sequencing, gene expression is measured over a bulk of cells. Thus, it is hard to study the heterogeneity of cells and characterize rare cell types such as stem cells and cancer cells [3]. Encouragingly, the recent breakthrough in single cell RNA sequencing (scRNA-seq) enables us to screen heterogeneous cells [4, 5].

One important task in scRNA-seq data analysis is to infer the categories of cells, which is crucial to elucidate cell types and understand cell functions. Clustering is a widely used solution to this task. However, scRNA-seq data characteristics of high noise level, dropout events (i.e. expressed genes that are fail to be detected) and high dimensionality complicate this task [6]. By far, a number of clustering methods have been developed for scRNA-seq data. For example, Prabhakaran et al. proposed the BISCUIT method, which clusters scRNA-seq data by incorporating parameters of technical variation into a Hierarchical Dirichlet Process mixture model [7]. Lin et al. developed an ultrafast algorithm CIDR that takes dropout events into account with a simple implicit imputation approach [8]. By combining multiple clustering solutions, a consensus clustering approach SC3 was designed to cluster scRNA-seq data [9]. DIMM-SC was specifically proposed for processing droplet-based scRNA-Seq data, which is based on the Dirichlet mixture model [10]. To handle the challenge of high dimensionality in scRNA-seq data, dimension reduction techniques were widely used. For example, pcaReduce integrated principal component analysis (PCA) with an agglomerative clustering method [11]. Shao et al. adapted nonnegative matrix factorization (NMF) to identify subpopulations in scRNA-seq data and showed that NMF outperforms PCA in accuracy and robustness [12]. CellTree applies latent dirichlet allocation (LDA) and produces the tree structure of single cells [13]. As shared nearest neighbor (SNN) has been demonstrated more stable and robust for high-dimensional data than traditional distance metrics, Chen et al. proposed SNNCliq, which identifies clusters by a quasi-clique-based clustering algorithm on a SNN graph [14], while the Seurat method finds clusters of cells by a modularity optimization-based clustering algorithm on a SNN graph [15]. Other methods like GiniClust and RaceID were developed to solve specific clustering task of rare cell type detection [16, 17]. Recently, deep learning-based methods such as scVI and SAUCIE were proposed to analyze scRNA-seq data [18, 19].

Although significant progress has been made in clustering scRNA-seq data, existing clustering methods still suffer from various limitations and there is much space to improve clustering accuracy. Most existing methods require to pre-specify the number of clusters to be output, which is impractical or even impossible for complex and large-scale datasets. Some methods such as probability model-based or deep learning-based methods, are sensitive to parameters and difficult to implement in practice. As for graph theory-based approaches, they usually use sparse SNN graphs, which tends to obtain excessive amounts of sub-graphs, resulting in low clustering accuracy. In summary, the rapidly increasing of scRNA-seq data and the drawbacks of existing methods call for novel scRNA-seq data clustering solutions.

In this paper, we propose a new and effective approach for scRNA-seq data clustering. It is a two-step clustering method called *TSC* — the abbreviation of *T*wo-*S*tep *C*lustering. That is, after splitting all cells into *core cells* that are closely connected with their neighbors and possibly lie around the centers of the underlying clusters, and *non-core cells* that are less closely connected with their neighbors and possibly located in the boundary areas of the clusters, we first group the core cells by hierarchical clustering (*the first step*) and then assign the non-core cells into the corresponding nearest clusters (*the second step*).

Technically, our method features in the following aspects: 1) we employ a “two-step clustering” strategy, which aims to cluster core cells and non-core cells separately, thus alleviate the negative impact of non-core cells (or boundary cells) on clustering accuracy. 2) In data-preprocessing, we propose the right-skewed coefficient (RSC) to measure the degree of right-skewedness in scRNA-seq data, and with RSC we can correctly determine whether or not to conduct Log-transformation on the data. 3) We apply random walk to represent the relationship between cells and define the random walk distance, which is used in hierarchical clustering of scRNA-seq data. 4) To generate reliable cell graph, we consider five simialrity/distance metrics, including three distance metrics and two correlation metrics. 5) We adopt an effective criterion to automatically determine the number of clusters to generate.

To evaluate the proposed method, we conduct extensive experiments on 12 real scRNA-seq datasets. Our experimental results show that the proposed method outperforms several state of the art methods in clustering scRNA-seq data.

## Results

In this section, we evaluate TSC in clustering scRNA-seq data. First, we introduce 12 publicly available scRNA-seq datasets and clustering evaluation metric. Then, we compare the effects of similarity/distance metrics applied in TSC on clustering accuracy. Third, we compare the clustering results of TSC with other methods. Fourth, we present the advantage of two-step clustering. Finally, we discuss the effectiveness of Log-transformation.

### Datasets and performance metric

We collected twelve real and publicly available scRNA-seq datasets from published papers. These datasets mainly contain scRNA-seq data about different cell types of mouse embryos, mouse cortex and mouse distal lung epithelium. The datasets have been widely used in evaluating existing scRNA-seq data clustering methods.

Table 1 presents the statistical information of these datasets, including the number of cells, clusters and genes and their sequencing protocols. Datasets are named by the accession numbers provided in the original publications. We can note that these datasets range in size from dozens to thousands, with more than 14,000 genes/transcripts. The number of cell types varies from 3 to 14. Units of gene/transcript levels include FPKM (Fragments Per Kilobase of exon model per Million mapped reads), CPM (Counts of exon model per Million mapped reads) and UMI (Unique Molecule Identifier). Specifically, UMI uses a direct measurement of transcript copies for each transcript [20], while FPKM and CPM normalize the raw read counts based on sequencing depth and gene length. In addition, these scRNA-seq data were generated from some representative sequencing platforms, such as Smart-seq [21], SMARTer [22], Smart-Seq2 [23, 24] and inDrop [25].

**Table 1:** A summary of 12 sc-RNAseq datasets

Datasets	#Cells	#Clusters	#Genes	Unit	Protocol
GSE59892 [26]	49	3	25737	FPKM	Smart-seq
GSE52583 [27]	80	5	23837	FPKM	SMARTer
E-MTAB-3321 [28]	124	5	28223	CPM	Smart-Seq2
E-MTAB-2600 [29]	704	3	21231	CPM	Smart-Seq2
GSE71585 [30]	1809	7	24057	Count	SMARTer
GSE65525 [25]	2717	4	24175	UMI	inDrop
GSM2230757 [31]	1937	14	20125	UMI	inDrop
GSM2230758 [31]	1724	14	20125	UMI	inDrop
GSM2230759 [31]	3605	14	20125	UMI	inDrop
GSM2230760 [31]	1303	14	20125	UMI	inDrop
GSM2230761 [31]	822	13	14878	UMI	inDrop
GSM2230762 [31]	1064	13	14878	UMI	inDrop

In our experiments, we use Adjusted Rand Index (ARI) to measure the clustering performance. Given the ground truth class assignments $labels\_true$ and the predicted class assignments $labels\_predict$, ARI measures the similarity of these two assignments [32]. Concretely, the overlapping between two assignments can be summarized as a contingency table, which reports the intersection cardinality of each true-predicted cluster pair. ARI is calculated as follows:

1 $$\begin{aligned} ARI= \frac{\sum _{ij}\left( {\begin{array}{c}t_{ij}\\ 2\end{array}}\right) - \left[ \sum _{i}\left( {\begin{array}{c}a_i\\ 2\end{array}}\right) \sum _{j}\left( {\begin{array}{c}b_j\\ 2\end{array}}\right) \right] / \left( {\begin{array}{c}m\\ 2\end{array}}\right) }{\frac{1}{2}\left[ \sum _{i}{}\left( {\begin{array}{c}a_i\\ 2\end{array}}\right) + \sum _{j}{}\left( {\begin{array}{c}b_j\\ 2\end{array}}\right) \right] - \left[ \sum _{i}\left( {\begin{array}{c}a_i\\ 2\end{array}}\right) \sum _{j}\left( {\begin{array}{c}b_j\\ 2\end{array}}\right) \right] / \left( {\begin{array}{c}m\\ 2\end{array}}\right) } \end{aligned}$$

where *m* is the number of cells totally in the dataset, $t_{ij}$ is the value at the $i^{th}$-row and the $j^{th}$-column in the contingency table, $a_i$ is the sum of the $i^{th}$-row of the contingency table, $b_j$ is the sum of the $j^{th}$-column of the contingency table, and () denotes a binomial coefficient. ARI ranges from -1 to 1, where a negative value means mismatch and ‘1’ indicates a perfect match. Other three commonly used clustering performance evaluation metrics are also applied in this paper, including Normalized Mutual Information (NMI) [33], Adjusted Mutual Information (AMI) [34] and Accuracy (Acc) [35].

### Comparison among different similarity/distance metrics

Here we compare the performance when using the five different similarity/distance metrics: ED (Euclidean distance), MD (Manhattan distance), PCC (Pearson correlation coefficient), SCC (Spearman correlation coefficient) and SNN (shared nearest neighbors). We denote the methods used these metrics as TSC$_{ED}$, TSC$_{MD}$, TSC$_{PCC}$, TSC$_{SCC}$ and TSC$_{SNN}$, respectively.

**Fig. 1:**
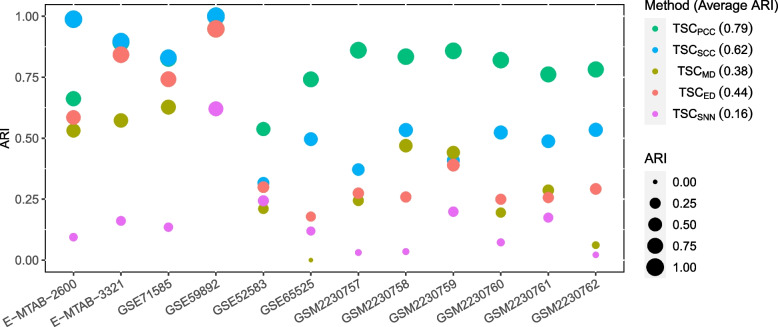
Performance comparison among the 5 similarity/distance metrics. The value in the parentheses following each method’s name in the legend is the average ARI

Figure 1 shows the ARI results on the 12 datasets. We can see that TSC$_{SCC}$ achieves the best results on the first four datasets, and TSC$_{PCC}$ performs best on the last nine datasets. Their average ARI values over the 12 datasets are 0.62 and 0.79 respectively, larger than those of the other three metrics. Overall, TSC$_{ED}$ and TSC$_{MD}$ are in the middle, and TSC$_{SNN}$ performs worst. So in the remaining experiments, we consider only TSC$_{SCC}$ and TSC$_{PCC}$.

### Comparison with existing methods

**Fig. 2:**
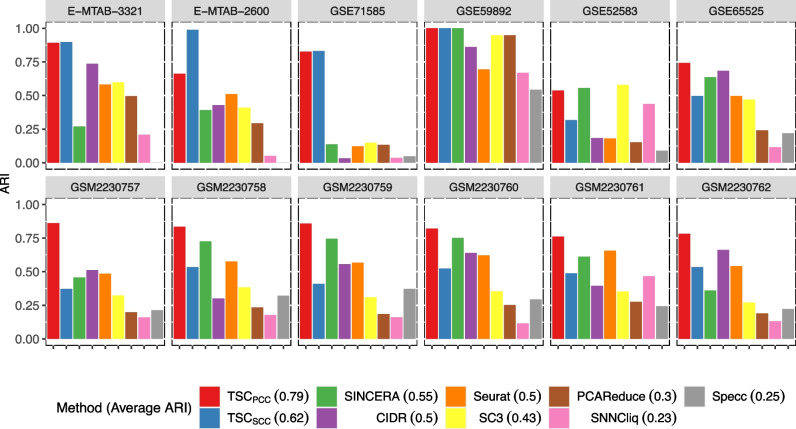
Performance comparison with seven existing methods on 12 datasets. The value in the parentheses following each method’s name in the legend is the average ARI

Here, we compare our method with six existing methods, including SC3 [9], CIDR [8], SINCERA [36], pcaReduce [11], Seurat [15] and SNNCliq [14]. They represent the state of the art of scRNA-seq data clustering [37, 38]. In addition, we also applied spectral clustering (a classical graph-based clustering method) to the scRNA-seq data. The ARI results are illustrated in Fig. 2, where the value in the parentheses following each method’s name in the legend is the average ARI over the 12 datasets.

From Fig. 2, we can see that TSC$_{PCC}$ outperforms the others on 8 of the 12 datasets, and TSC$_{SCC}$ performs best on 4 of the 12 datasets. They achieve 0.79 and 0.62 of average ARI over the 12 datasets respectively, which are much higher than those of the 6 existing methods. This result validates the advantage of our method over the existing ones. For the existing methods, SINCERA performs best on average, followed by Seurat, CIDR, SC3, pcaReduce and spectral clustering. SNNCliq performs worst. Results of the other three clustering performance metrics show similar trends as that of ARI, which are presented in the Additional file (Additional file 1: Table S1).

### Advantage of two-step clustering

Our TSC method adopts a “two-step clustering” strategy. To further demonstrate the advantage of our method, here we compare the performance of our “two-step clustering” strategy and that of the “one-step clustering” strategy. In the “one-step clustering” strategy, we do not split cells to core cells and non-core cells, instead we directly cluster all cells. Note that in the “one-step clustering” strategy, we use similar data processing strategy, random walk distance and hierarchical clustering as in the “two-step clustering” strategy. Both use PCC in graph construction for random walk. The results are presented in Table 2. Here, the 2nd column (“ARI-1Step”) presents the ARI results of “one-step clustering”. The 3rd column and the 4th column give the ARI results of TSC$\_PCC$, but the former “ARI-2Steps-core” indicates the ARI computed only on core cells, and the latter “ARI-2Steps” is the ARI computed on all cells.

**Table 2:** Comparison between one-step clustering and two-step clustering

Dataset	ARI-1Step	ARI-2Steps-core	ARI-2Steps
GSE59892	1	1	1
E-MTAB-3321	0.63	0.87	0.89
E-MTAB-2600	0.38	0.61	0.66
GSE52583	0.01	0.61	0.53
GSE65525	0.41	0.74	0.74
GSE71585	0.27	1	0.82
GSM2230757	0.62	0.86	0.86
GSM2230758	0.76	0.87	0.83
GSM2230759	0.87	0.89	0.85
GSM2230760	0.79	0.87	0.82
GSM2230761	0.67	0.76	0.76
GSM2230762	0.80	0.92	0.78
Average ARI	0.60	0.83	0.79

From Table 2, we can see that our “two-step clustering” strategy is more effective than the “one-step clustering” strategy on 10 of the 12 datasets. On average, the ARI of our method is 28% higher than that of the “one-step clustering” strategy. Furthermore, by comparing the results of “ARI-2steps-core” and “ARI-2steps” over 12 datasets, we can find that the ARI of “ARI-2steps-core” is higher than that of “ARI-2steps” on all 12 datasets. This is consistent with our expectation that core cells are easier to be clustered than non-core cells.

### Effectiveness of Log-transformation

TSC will examine whether or not to perform Log-transformation in data preprocessing. We propose RSC as the criterion of Log-transformation. To evaluate the effectiveness of RSC, in Table 3 we present the RSC values and the corresponding ARI values of TSC$_{PCC}$ on the 12 scRNA-seq datasets. The 3rd/4th column is the ARI values of TSC$_{PCC}$ without/with Log-transformation.

**Table 3:** ARI comparison of TSC$_{PCC}$ with/without Log-transformation

Dataset	RSC	ARI-NoLog	ARI-Log
E-MTAB-3321	1.80	0.22	**0.89**
E-MTAB-2600	1.31	0.08	**0.66**
GSE59892	1.29	0.57	**1**
GSE52583	1.08	0.50	**0.53**
GSE71585	0.83	0.69	**0.82**
GSM2230759	0.49	**0.85**	0.71
GSM2230758	0.48	**0.83**	0.78
GSM2230761	0.46	**0.76**	0.48
GSM2230762	0.43	**0.78**	0.78
GSM2230760	0.42	**0.82**	0.78
GSE65525	0.39	**0.74**	0.40
GSM2230757	0.34	**0.86**	0.67

As shown in Table 3, we can see that the first five datasets (from E-MTAB-3321 to GSE71585) have relatively large RSC ($> 0.80$), and their ARI values when using Log-transformation are much larger than that when not using Log-transformation. On the contrary, for the other seven datasets, they have relatively small RSC ($<0.5$), and their ARI values when not using Log-transformation are much larger than that when using Log-transformation.

In summary, from Table 3 we can conclude that 1) RSC is effective in correctly deciding whether or not to perform Log-transformation; 2) When Log-transformation is properly performed according to our RSC criterion, significant improvement on ARI can be achieved.

### Effects of parameters in TSC

To select core cells, we adopted a threshold to filter the edges from the fully connected graph. Here, we check the clustering performance of TSC under four cases, i.e., keeping 25% , 50%, 75% and 100% the edges in the fully connected graph. From the results shown in the Additional file (Additional file 2: Fig. S1), we can see that TSC achieves the best clustering accuracy on the twelve datasets when keeping 25% edges in the fully connected graph.

To calculate the distance between cells, we perform random walk on the cell graph, in which the step size (parameter *t*) plays a key role in cells’ similarity evaluation. Here, we analyze the effect of parameter *t* on the clustering performance of *TSC*. Concretely, we evaluate the robustness of *TSC* to *t* as follows: changing *t*’s value from 2 to 15, and evaluating the clustering performance by ARI, the results are shown in the Additional file (Additional file 3: Fig. S2). We can see that *TSC* has relatively stable ARI when *t* increases from 2 to 15 on most of the datasets, and by setting *t* to 4 or 6 can get better performance.

## Discussion

scRNA-seq clustering is the most direct and effective method to identify novel cell types and characterize the heterogeneous cell populations. Here, we introduce *TSC*, a novel two-step clustering method, to improve the clustering accuracy. To create a graph for core cells, we considered five different similarity/distance metrics. However, each metric owns its advantages, and it is not sufficient to choose one metric to measure the similarity between cells. For future work, we will try to improve cell graph construction by integrating multiple similarity/distance measurements to make the graphs more reliable, thus further boost clustering performance. On the other hand, considering that deep learning is effective in processing big data, we will also explore new deep learning models for effectively clustering scRNA-seq data. Last but not least, considering that annotated scRNA-seq data are much less than raw data without annotations, we will also intend to extend our *TSC* framework to large datasets by exploring semi-supervised strategies.

## Conclusion

In this paper, we develop a new and effective scRNA-seq data clustering method *TSC*, which adopts a two-step clustering strategy, by first splitting all cells into core cells and non-core cells. Then, the core cells are clustered by hierarchical clustering with random walk distance, and the non-core cells are finally assigned to the clusters according to their distances to these clusters. With the two-step clustering strategy, *TSC* is able to guarantee the clustering accuracy of core cells and improve the overall accuracy subsequently. In addition, *TSC* does not need to specify the number of clusters, but determines the cluster number automatically. Moreover, we design the *RSC* criterion to determine whether or not to perform Log-transformation on data before clustering. Extensive experiments on 12 real datasets show that the proposed method outperforms the state of the art methods in scRNA-seq data clustering analysis. In addition, our experiments also show that 1) the two-step clustering strategy is much better than the one-step clustering strategy (directly clustering all cells); 2) The proposed *RSC* criterion is effective in deciding whether or not to perform Log-transformation on scRNA-seq data; 3) PCC and SCC are more effective in constructing cell graphs for clustering than the other three metrics ED, MD and SNN.

## Methods

**Fig. 3:**
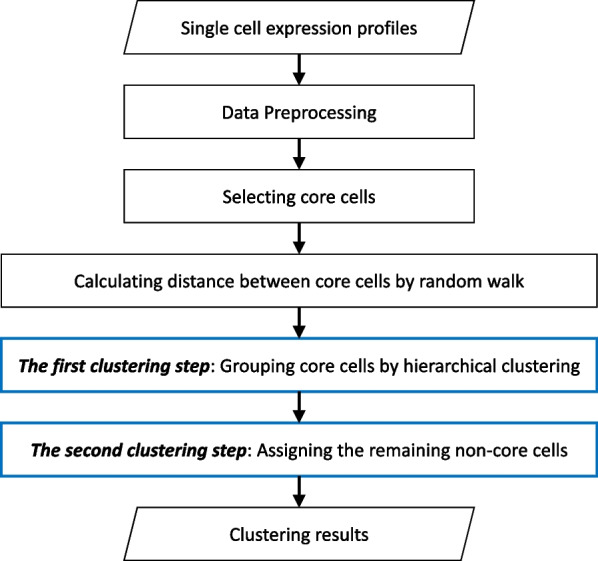
The pipeline of TSC

In this section, we describe the *TSC* method in detail. Figure 3 illustrates the pipeline of *TSC*, which consists of four major steps: 1) Data preprocessing; 2) Selecting core cells; 3) Calculating distance between core cells by random walk; 4) Grouping core cells by hierarchical clustering (*the first clustering step*); (5) Assigning the remaining non-core cells to the corresponding nearest clusters (*the second clustering step*).

In what follows, we give the technical detail of each module above.

### Data preprocessing

Since features with excessive amounts of 0 value are not informative for clustering, we first remove genes/transcripts that express (expression value >0) in less than 2% of cells. Actually, a small change to this percentage threshold does not significantly impact clustering result [9].

In scRNA-seq data, the expression levels of different genes vary greatly, which leads to the right-skewed distribution phenomena, i.e., the mean is greater than the median. Thus, the similarity or distance between cells would be largely determined by the genes with large values. Many scRNA-seq clustering approaches employ Log-transformation to handle right-skewed distribution. However, it is improper to perform Log-transformation on data not fitting right-skewed distribution. Otherwise, the difference between genes will be distorted. To solve this problem, we define a *right-skewed coefficient* (*RSC*) to measure the degree of right-skewness of data as follows:

2 $$\begin{aligned} RSC = \frac{\sum _{i=1, g_i^{max} \ge \mu }^{l} (g_i^{max}-\mu )}{l * \mu } \end{aligned}$$

where $g_i^{max}$ is the maximum expression value of gene *i*, $\mu$ is the average of all genes’ maximum values, and *l* is the number of genes whose maximum expression values are larger than $\mu$. *RSC* indicates the average deviation of data points that lie in the right of mean. The larger *RSC* is, the more the data are right-skewed. In this paper, when *RSC* is greater than 0.8, we think that the data are heavily right-skewed and Log-transformation is performed. To eliminate the effect of outliers, we remove genes that do not fall in [*Q*1-1.5*IQR, *Q*3+1.5*IQR] before computing *RSC* [39]. Here, *Q*1 and *Q*3 are the first and the third quartile of all genes’ maximum values, and the *interquartile range* (IQR) is (*Q*3-*Q*1).

### Selecting core cells

Given a scRNA-seq dataset, we find the *core cells* by first constructing a fully-connected weighted graph $G_c$ where each node corresponds to a cell and each edge-weight represents the *similarity* between the two respective cells.

Usually, the *similarity* between two cells can also evaluated by the difference between 1 and their corresponding *distance* when the distance is normalized into [0, 1]. So we can treat *similarity* and *distance* equally. We consider five similarity/distance measures: Euclidean distance (ED), Manhattan distance (MD), Pearson correlation coefficient (PCC), Spearman correlation coefficient (SCC) and shared nearest neighbors (SNN) [40]. ED and MD are commonly used distance measurements. PCC and SCC range from -1 to 1, we use only the positive values. SNN is also called second-order distance, which measures the similarity between two samples based on their shared neighbors.

Then, we set a similarity threshold $s_c$. In the graph $G_c$, we discard all the edges whose weights are less than $s_c$. The remaining edges and the nodes connected by any of these remaining edges form a new graph $G_{cc}$. We call the nodes in $G_{cc}$ *core nodes* as they are relatively close to their neighbors and possibly lie around the centers of the underlying cell clusters. Thus, the cells corresponding to the core nodes are *core cells*, and we call $G_{cc}$ core-cell graph. On the other hand, we call the remaining nodes *non-core nodes*, and the corresponding cells *non-core cells*. Non-core nodes are not close to their neighbors as the similarity values between them and their neighbors are less than $s_c$. So they may be located in the boundary areas of the underlying clusters.

As a rule of thumb, we choose $s_c$ such that the number of edges in $G_{cc}$ is around 25% of the total number of edges in $G_c$.

#### Calculating distance between core cells by random walk

To calculate the distance between any two core cells, we perform random walk on the core-cell graph $G_{cc}$ constructed above. The random walk process is as follows: Given the transition matrix *M* where $M_{ij}=\frac{w_{ij}}{Deg(i)}$, ${Deg(i)}=\sum _{j=1}^{n_i}w_{ij}$, $n_i$ means the number of neighbors of cell *i*, $w_{ij}$ is the similarity between cell *i* and cell *j*. Suppose there are *n* nodes in $G_{cc}$. If a walker starts from node (or cell) *i*, then the initial probability $P_{i.}^0$ is set as a *n*-dimension vector with only the $i^{th}$ dimension value being 1 and the others being 0. As the walker goes on the graph, the vector of probability is updated according to $P^{t+1}={M^T}*P^t$ where $P_{ij}^{t}$ is the probability of the walker going from node *i* to node *j* in *t* steps. It has been shown that if *t* becomes infinity, the probability $P_{ij}^{t}$ depends only on the degree of node *j*. Therefore, it is crucial to choose the value of *t*: too short will not be enough to capture the graph’s topological information, while too long will result in a stationary distribution. In our experiments, we set *t* = 4, which is empirically advised by previous study [41].

For cell *i*, we can obtain a vector of walking probability starting from it. The random walk distance $d_{ij}$ between cell *i* and cell *j* is defined as below:

3 $$\begin{aligned} d_{i j}=\sqrt{\sum\nolimits _{k=1}^{n}\frac{{(P_{ik}^t-P_{jk}^t)}^{2}}{Deg(k)}}. \end{aligned}$$

#### Grouping core cells by hierarchical clustering

We employ bottom-up hierarchical clustering to cluster the core cells. That is, first treat each core cell as a cluster, and then merge the nearest cluster pairs iteratively. The distance between two cells is calculated by Eq. (3). The distance $d_{Ck}$ between cell *k* and cluster *C* and the distance $d_{C_iC_j}$ between cluster $C_i$ and cluster $C_j$ are defined as follows:

4 $$\begin{aligned} d_{Ck}=\frac{1}{\left| C \right| }\sum\nolimits _{i\epsilon C}P_{ik}^t, \end{aligned}$$

5 $$\begin{aligned} d_{C_i C_j}=\sqrt{\sum\nolimits _{k=1}^{n}\frac{{(d_{C_ik}-d_{C_jk})}^{2}}{Deg(k)}} \end{aligned}$$

where $\left| C \right|$ indicates the number of cells in cluster *C*. One important issue in hierarchical clustering is the criteria for selecting two clusters to merge each time. Here, we adopt the strategy from the Wards method [42]. The change of the average intra-cluster distance before and after the merging of cluster $C_i$ and cluster $C_j$ is evaluated as follows:

6 $$\begin{aligned} \Delta \sigma (C_i,C_j)=\frac{1}{n}\left( \sum\nolimits _{k\in C_u}d_{C_uk}^2 - \sum\nolimits _{k\in C_i}d_{C_ik}^2 -\sum\nolimits _{k\in C_j}d_{C_jk}^2 \right) \end{aligned}$$

where $C_u=C_i \cup C_j$. We select the two clusters with the smallest value of $\Delta \sigma$ to merge each time.

Another important issue is to determine the number of clusters to be generated, we use the criteria introduced in [41]. First, evaluating the average intra-cluster distance $\sigma _K$ of *K* clusters as follows:

7 $$\begin{aligned} \sigma _K=\frac{1}{n}\sum\nolimits _{k=1}^{K}\sum\nolimits _{i \in C_k} d_{C_{k}i}^2 \end{aligned}$$

where $C_{k}$ means the $k^{th}$ cluster. Then, calculating the change of the average intra-cluster distance when the number of clusters increases from *K* to $K+1$ by

8 $$\begin{aligned} \eta _{K}=\frac{\sigma _{K+1}-\sigma _{K}}{\sigma _{K}-\sigma _{K-1}}. \end{aligned}$$

The optimal number *K* of clusters is that with the maximum value of $\eta _{K}$.

### Assigning the non-core cells

After clustering the core cells, we get *K* clusters. To assign the non-core cells to the generated clusters, we first evaluate the center of each cluster as follows:

9 $$\begin{aligned} c_{kj}=\frac{\sum _{x_c \in \chi _k} x_{cj}}{\left| \chi _k \right| } \end{aligned}$$

where $c_{kj}$ is the value in the $j^{th}$ dimension of the center vector of cluster *k*, $x_{cj}$ is the expression value of the $j^{th}$ gene of core cell *c*, $\chi _k$ is the set of core cells in cluster *k* and $\left| \chi _k \right|$ indicates the number of core cells in cluster *k*.

For each non-core cell, we then calculate its distance to the center of each cluster, and assign it to the cluster whose center is nearest to the cell.

### Supplementary information

**Additional file 1: Table S1. **Clustering performance evaluation with four metrics.

**Additional file 2: Figure S1. **ARI vs. edge filtering threshold. For the sub-graph of each database, the horizontal coordinate corresponds to four cases: the number of edges in the graph is N_e_ , 3/4 N_e_, 1/2Ne and 1/4N_e_ , where N_e_ indicates the number of edges in the fully connected graph. Curves of different colors represent results of TSC with different similarity/distance measurements.

**Additional file 3: Figure S2.** ARI of TSC_PCC_ vs. parameter *t*. The horizontal coordinate corresponds to the value of parameter *t*, and curves of different colors correspond to the results on different data sets.

## Data Availability

The datasets used and/or analysed in this study are available from the corresponding articles. Ten datasets are available in the GEO repository with accession number GSE59892, GSE52583, GSE71585, GSE65525 and GSE84133 (including the datasets from GSM2230757 to GSM2230762) (https://www.ncbi.nlm.nih.gov/geo/query/acc.cgi?acc=GSE59892, https://www.ncbi.nlm.nih.gov/geo/query/acc.cgi?acc=GSE52583, https://www.ncbi.nlm.nih.gov/geo/query/acc.cgi?acc=GSE71585, https://www.ncbi.nlm.nih.gov/geo/query/acc.cgi?acc=GSE65525), https://www.ncbi.nlm.nih.gov/geo/query/acc.cgi?acc=GSE84133). Two datasets are available in the ArrayExpress repository with accession number E-MTAB-3321 and E-MTAB-2600 (https://www.ebi.ac.uk/arrayexpress/experiments/E-MTAB-3321/, https://www.ebi.ac.uk/arrayexpress/experiments/E-MTAB-2600/). The source code of TSC is available at https://github.com/LiRuiyi-raptor/TSC_Project.

## Abbreviations

scRNA-seq: single cell RNA-sequencing
TSC: Two-Step Clustering
SC3: Single-cell consensus clustering
SINCERA: Single cell RNA-seq profiling analysis
CIDR: Clustering through imputation and dimensionality reduction
RaceID: Rare cell type identification
DIMM-SC: Dirichlet mixture model for clustering droplet-based single cell transcriptomic data
NMF: Nonnegative matrix factorization
ED: Euclidean distance
MD: Manhattan distance
PCC: Pearson correlation coefficient
SCC: Spearman correlation coefficient
SNN: Shared nearest neighbors
FPKM: Fragments per kilobase of exon model per million mapped reads
CPM: Counts of exon model per million mapped reads
UMI: Unique molecule identifier
ARI: Adjusted rand index
