# Supplementary material for: A new and effective two-step clustering approach for single cell RNA sequencing data
Source: BMC Genomics. 2023 Nov 9;23(Suppl 6):864. doi: 10.1186/s12864-023-09577-x (PMC10636845; doi:10.1186/s12864-023-09577-x)
Supplement: Supplementary file 1 — Additional file 1: Table S1. Clustering performance evaluation with four metrics. [file 12864_2023_9577_MOESM1_ESM.pdf]

Additional file1

Table S1: Clustering performance evaluation with four metrics.

Dataset	Methods	Clustering performance evaluation			
		ARI	NMI	AMI	ACC
GSE59892	TSCpcc	1	1	1	1
	TSCscc	1	1	1	1
	SC3	0.9483	0.9293	0.9261	0.9796
	CIDR	0.8606	0.886	0.8763	0.898
	SINCERA	1	1	1	1
	pcaReduce	0.9483	0.9293	0.9261	0.9796
	Seurat	0.6951	0.7867	0.7813	0.8163
	SNNCliq	0.6686	0.7424	0.7154	0.7551
	Spectral clustering	0.5427	0.5575	0.5373	0.8367
E-MTAB-3321	TSCpcc	0.8926	0.8642	0.8505	0.7984
	TSCscc	0.8974	0.8787	0.8701	0.8226
	SC3	0.5973	0.8009	0.7876	0.629
	CIDR	0.7355	0.785	0.7655	0.7258
	SINCERA	0.2692	0.5333	0.5024	0.4919
	pcaReduce	0.4954	0.6293	0.6038	0.6371
	Seurat	0.5821	0.7595	0.7487	0.7097
	SNNCliq	0.2085	0.5815	0.5159	0.371
E-MTAB-2600	TSCpcc	0.662	0.7401	0.7391	0.8438
	TSCscc	0.988	0.979	0.9789	0.9957
	SC3	0.4089	0.6533	0.6505	0.4588
	CIDR	0.4278	0.5685	0.5663	0.5966
	SINCERA	0.3916	0.4604	0.4544	0.642
	pcaReduce	0.2937	0.5315	0.5278	0.3722
	Seurat	0.5098	0.695	0.6932	0.571
	SNNCliq	0.0515	0.252	0.2442	0.4375
	TSCpcc	0.5375	0.5178	0.448	0.7125
	TSCscc	0.3166	0.32	0.2553	0.575

GSE52583

	SC3	0.5787	0.6156	0.5863	0.7375
	CIDR	0.1834	0.3718	0.3054	0.4875
	SINCERA	0.5567	0.5392	0.5047	0.7125
	pcaReduce	0.1518	0.2472	0.1825	0.575
	Seurat	0.1796	0.1413	0.1168	0.525
	SNNCliq	0.4379	0.4379	0.373	0.7
	Spectral clustering	0.0889	0.2842	0.222	0.3375
GSE65525	TSCpcc	0.7413	0.7117	0.7107	0.8086
	TSCscc	0.4961	0.6514	0.6512	0.5933
	SC3	0.4701	0.699	0.6973	0.5329
	CIDR	0.6833	0.6617	0.6608	0.7332
	SINCERA	0.6363	0.6955	0.6929	0.7858
	pcaReduce	0.2419	0.5708	0.5687	0.2573
	Seurat	0.4969	0.7186	0.7177	0.5506
	SNNCliq	0.115	0.3334	0.2918	0.1925
	Spectral clustering	0.2193	0.2859	0.285	0.5046
GSM2230757	TSCpcc	0.8607	0.8105	0.8082	0.8296
	TSCscc	0.3714	0.5017	0.499	0.5694
	SC3	0.3226	0.706	0.6965	0.5416
	CIDR	0.5122	0.5518	0.5496	0.6458
	SINCERA	0.4569	0.6749	0.6654	0.5983
	pcaReduce	0.1975	0.6173	0.6051	0.3583
	Seurat	0.485	0.8093	0.8056	0.6613
	SNNCliq	0.1588	0.5745	0.5348	0.35
	Spectral clustering	0.2133	0.583	0.5744	0.3774
GSM2230758	TSCpcc	0.8344	0.7885	0.7863	0.8799
	TSCscc	0.5334	0.4861	0.4727	0.6995
	SC3	0.3844	0.7032	0.694	0.5093
	CIDR	0.3013	0.4416	0.4325	0.54
	SINCERA	0.7255	0.784	0.7777	0.8103
	pcaReduce	0.2333	0.6288	0.6168	0.3387
	Seurat	0.5755	0.7814	0.7772	0.5719
	SNNCliq	0.1768	0.5657	0.5271	0.3538

	Spectral clustering	0.3212	0.5827	0.5731	0.4623
GSM2230759	TSCpcc	0.8581	0.7825	0.7814	0.8865
	TSCscc	0.4087	0.5298	0.5278	0.5506
	SC3	0.3098	0.6659	0.6591	0.4122
	CIDR	0.5559	0.6505	0.6489	0.7517
	SINCERA	0.7452	0.7829	0.7789	0.7864
	pcaReduce	0.1843	0.5916	0.5833	0.2541
	Seurat	0.5671	0.7839	0.7817	0.6252
	SNNCliq	0.1608	0.5039	0.4579	0.2444
	Spectral clustering	0.3725	0.5965	0.5923	0.4802
GSM2230760	TSCpcc	0.8202	0.7799	0.7771	0.8979
	TSCscc	0.5234	0.5074	0.496	0.6761
	SC3	0.3548	0.6983	0.6888	0.4574
	CIDR	0.6388	0.6602	0.6575	0.7751
	SINCERA	0.7504	0.7761	0.7693	0.8312
	pcaReduce	0.2519	0.6272	0.6152	0.3361
	Seurat	0.6213	0.8032	0.7994	0.693
	SNNCliq	0.1156	0.5015	0.4476	0.2341
	Spectral clustering	0.2936	0.5737	0.5627	0.4459
GSM2230761	TSCpcc	0.7617	0.7625	0.752	0.8771
	TSCscc	0.4875	0.5635	0.5562	0.6448
	SC3	0.353	0.6814	0.6663	0.4623
	CIDR	0.395	0.5566	0.5383	0.5389
	SINCERA	0.6108	0.7458	0.7353	0.6618
	pcaReduce	0.2747	0.6217	0.6037	0.3929
	Seurat	0.6553	0.8028	0.7968	0.7251
	SNNCliq	0.4661	0.6392	0.6069	0.5414
	Spectral clustering	0.2427	0.4665	0.4432	0.4404
GSM2230762	TSCpcc	0.782	0.6629	0.6538	0.8327
	TSCscc	0.5343	0.5095	0.4903	0.6372
	SC3	0.2713	0.6239	0.6081	0.4662
	CIDR	0.6624	0.5998	0.592	0.7265
	SINCERA	0.3604	0.5622	0.5468	0.5461

	pcaReduce	0.1894	0.5697	0.5513	0.3393
	Seurat	0.5409	0.7683	0.7616	0.6523
	SNNCliq	0.1307	0.5208	0.4659	0.2914
	Spectral clustering	0.2224	0.5609	0.5455	0.3393
GSE71585	TSCpcc	0.8268	0.7452	0.7447	0.9097
	TSCscc	0.8303	0.8183	0.8165	0.8778
	SC3	0.1488	0.4575	0.4046	0.2421
	CIDR	0.0326	0.2085	0.1985	0.0912
	SINCERA	0.1365	0.4379	0.3947	0.2327
	pcaReduce	0.1332	0.4391	0.3768	0.2311
	Seurat	0.122	0.4096	0.3688	0.23
	SNNCliq	0.0355	0.2992	0.2157	0.1454
	Spectral clustering	0.048	0.1581	0.1513	0.3266
