# Supplementary figures and images for: A new and effective two-step clustering approach for single cell RNA sequencing data
Source: BMC Genomics. 2023 Nov 9;23(Suppl 6):864. doi: 10.1186/s12864-023-09577-x (PMC10636845; doi:10.1186/s12864-023-09577-x)

ARI

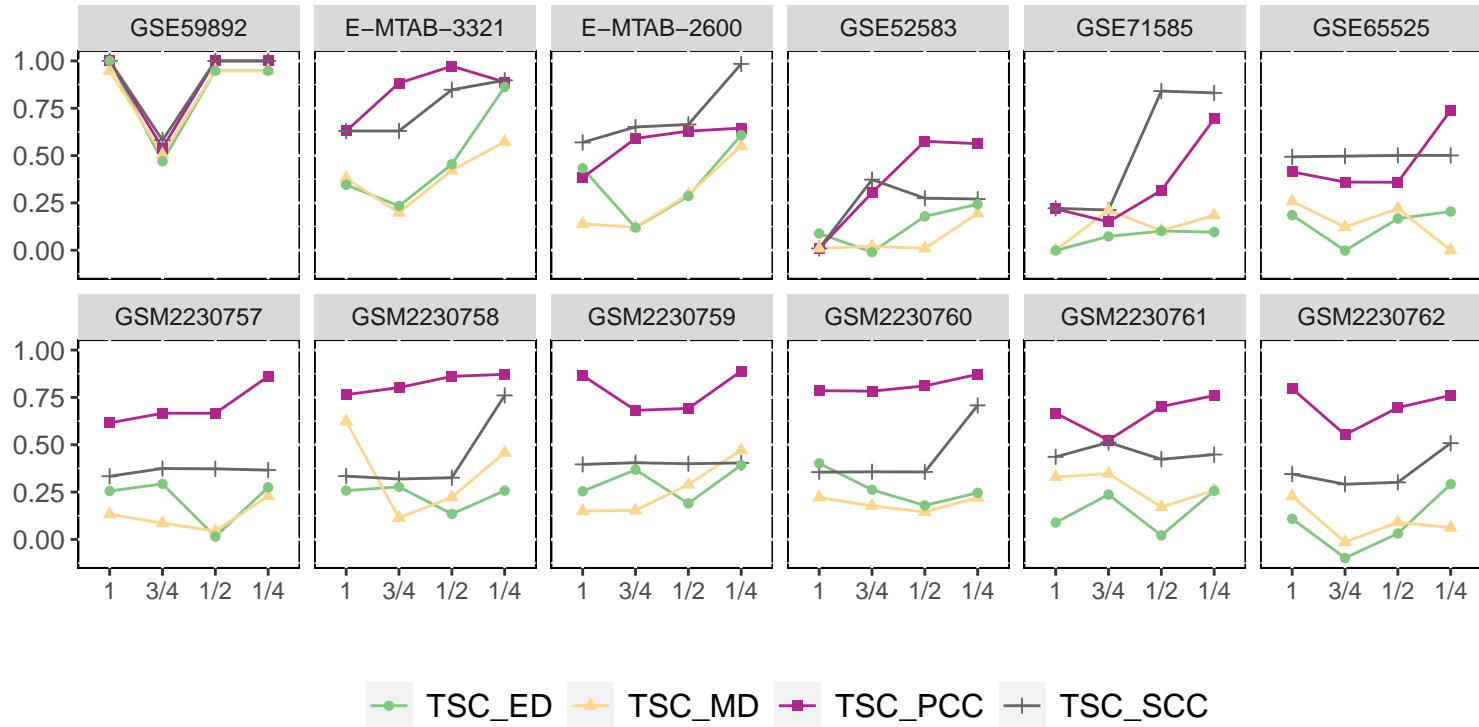

Supplement: Supplementary file 2 — Additional file 2: Figure S1. ARI vs. edge filtering threshold. For the sub-graph of each database, the horizontal coordinate corresponds to four cases: the number of edges in the graph is Ne , 3/4 Ne, 1/2Ne and 1/4Ne , where Ne indicates the number of edges in the fully connected graph. Curves of different colors represent results of TSC with different similarity/distance measurements. [file 12864_2023_9577_MOESM2_ESM.pdf]

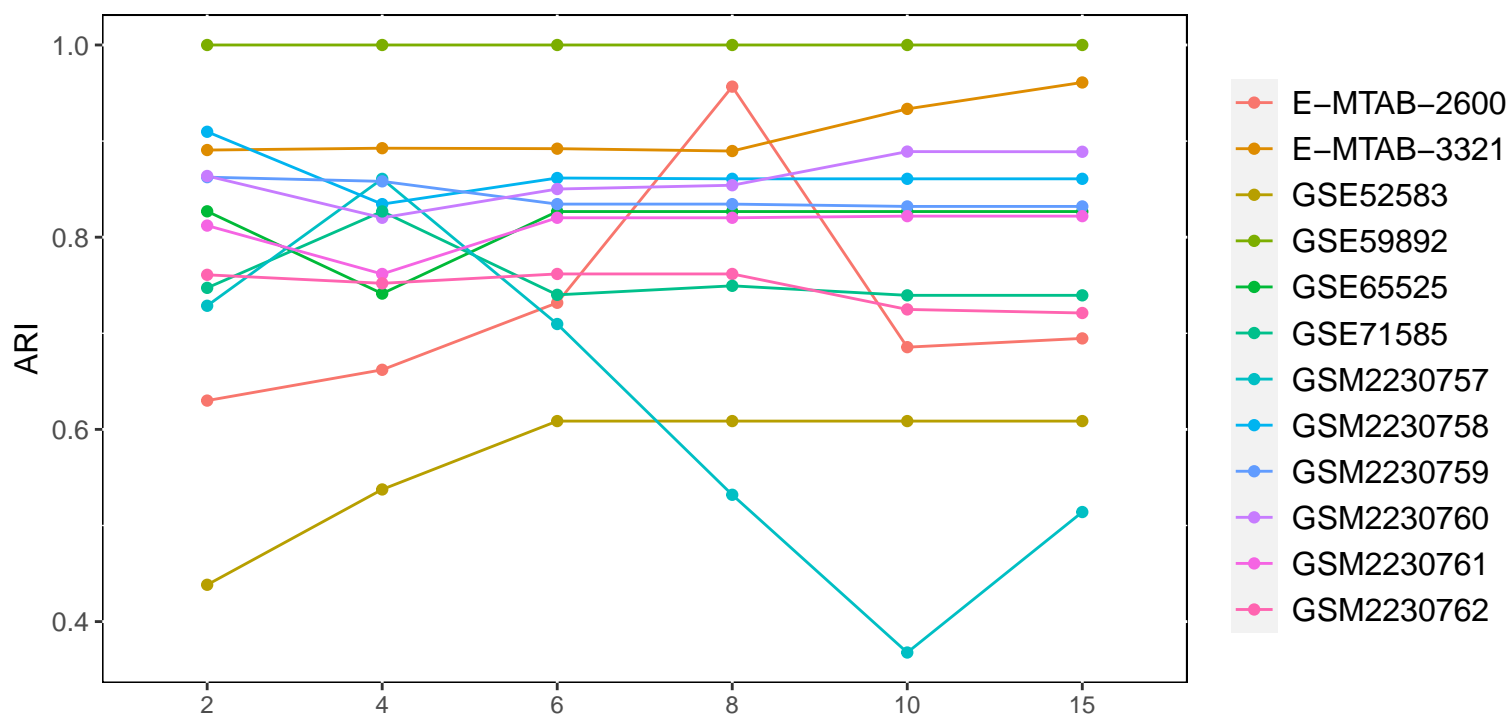

Supplement: Supplementary file 3 — Additional file 3: Figure S2. ARI of TSCPCC vs. parameter t. The horizontal coordinate corresponds to the value of parameter t, and curves of different colors correspond to the results on different data sets. [file 12864_2023_9577_MOESM3_ESM.pdf]
